# Green Chemistry Approach for Synthesis of Effective Anticancer Palladium Nanoparticles

**DOI:** 10.3390/molecules201219860

**Published:** 2015-12-15

**Authors:** Sangiliyandi Gurunathan, EunSu Kim, Jae Woong Han, Jung Hyun Park, Jin-Hoi Kim

**Affiliations:** Department of Animal Biotechnology, Konkuk University, 1 Hwayang-Dong, Gwangin-gu, Seoul 143-701, Korea; np-gennao@hanmail.net (E.S.K.); woong1211@naver.com (J.W.H.); saladinhyun@naver.com (J.H.P.)

**Keywords:** palladium nanoparticles, cell viability, ovarian cancer cells, apoptosis, anticancer activity, leaf extract

## Abstract

The purpose of this study was to design and synthesize Palladium nanoparticles (PdNPs) using an environmentally friendly approach and evaluate the *in vitro* efficacy of PdNPs in human ovarian cancer A2780 cells. Ultraviolet-Visible (UV-Vis) spectroscopy was used to monitor the conversion of Pd(II) ions to Pd(0)NPs. X-ray diffraction (XRD) revealed the crystallinity of the as-synthesized PdNPs and Fourier transform infrared spectroscopy (FTIR) further confirmed the role of the leaf extract of *Evolvulus alsinoides* as a reducing and stabilizing agent for the synthesis of PdNPs. Dynamic light scattering (DLS) and transmission electron microscopy (TEM) showed that the average size of the NPs was 5 nm. After a 24-h exposure to PdNPs, cell viability and light microscopy assays revealed the dose-dependent toxicity of the PdNPs. Furthermore, the dose-dependent cytotoxicity of the PdNPs was confirmed by lactate dehydrogenase (LDH), increased reactive oxygen species (ROS) generation, activation of PdNPs-induced autophagy, impairment of mitochondrial membrane potential (MMP), enhanced caspase-3 activity, and detection of TUNEL-positive cells. Our study demonstrates a single, simple, dependable and green approach for the synthesis of PdNPs using leaf extracts of *Evolvulus alsinoides.* Furthermore, the *in vitro* efficacy of PdNPs in human ovarian cancer cells suggests that it could be an effective therapeutic agent for cancer therapy.

## 1. Introduction

The synthesis of nanometer-sized noble metals has been the focus of much interest in academia and the industry due to their remarkable physical and chemical properties including catalytic, electronic, magnetic, optical, mechanical, and biological [1,2,3,4]. In addition, the physical and chemical properties of nanomaterials differ from those of bulk materials both quantitatively and qualitatively [1,2,3,4]. Among the numerous known nanomaterials, palladium nanoparticles (PdNPs) are a focus of great interest owing to their applicability as both heterogeneous and homogeneous catalysts, high surface-to-volume ratio, and high surface energy [5]. In addition, PdNPs have been used as catalysts in various coupling reactions such as Heck coupling [6], Suzuki coupling [7], and hydrogenation of allyl alcohols [8].

PdNPs are of great importance as catalytic materials and for numerous other applications such as hydrogen storage and sensing [9]. Pd is one of the most efficient metals used in catalysis [10,11] and has been widely studied in catalytic applications including hydrogenation [12], oxidation [13], carbon-carbon bond formation [14], and electrochemical reactions in fuel cells [15]. The applications of PdNPs in the biomedical field are currently limited. However, a few reports have suggested that PdNPs possess the antibacterial and antitumor activity. Recently, Fang *et al.* [16] reported a simple route for the preparation of Pd nanosheet-covered hollow mesoporous NPs for the combined chemotherapy and photothermal therapy of cancer cells. The combined therapy produces a synergistic effect with higher efficacy than the sum of the individual efficacies of chemotherapy and photothermal therapy. Elhusseiny and Hassan [17] reported that Pd complexes of polyamides containing sulfones showed the highest antibacterial and antifungal potency. Furthermore, PdNPs supported on mesoporous silica SBA-15 and MSU-2 showed high toxicities against five different human cancer cell lines [18]. On the other hand, numerous studies have reported the potential toxicity of PdNPs and their propensity to cause adverse health effects such as concentration-dependent cytotoxicity, apoptosis, and alterations in the release and expression of numerous cytokines [19,20,21,22,23,24,25].

The synthesis of PdNPs is indispensable for various electronic as well as biomedical and biotechnological applications and, therefore, different methods have been adopted including reduction [26], metal vaporization [27], sol-gel process [28], laser ablation [29], sonochemical [30], and biological methods [31]. Chemical methods usually appear to be simple but may cause extensive aggregation, which has been avoided by using stabilizers and supporting materials to obtain homogeneously distributed samples [32,33,34]. In addition, chemical methods require capping agents that may severely limit the catalytic activity [35] and are hazardous to the environment. Therefore, Sun *et al.* [35] demonstrated that the use of high-surface-area materials as supports is a promising strategy for the preparation of ultrafine and well-defined noble metal NPs. These supporting materials not only function as barriers to prevent the encapsulated NPs from coalescing but also improve the chemical and thermal stability and enhance the electrical conductivity of the functional materials [35].

To avoid the shortcomings of chemical methods, the use of green chemistry approaches has recently increased in the production of various types of nanomaterials such as Pd, silver, graphene, and gold [31,36,37,38]. In addition, biological methods are capable of controlling the shape and size of monodispersed NPs [38]. Numerous previous studies have reported the synthesis of PdNPs using various biological materials including coffee and tea extracts [31], *Cinnamomum zeylanicum* bark [39], banana peel extract [40], *Cinnamomum camphora* leaf broth [4], gum acacia [41], *Annona squamosa* peel extract [42], *Anacardium occidentale* [43], *Pulicaria glutinosa* extract [44], and xanthan gum [45]. Although there are reports of green synthesis of PdNPs using plant materials, it would be expedient to use unexplored biological materials in the preparation of scalable, stable, homogeneous, and dispersible colloidal PdNPs [44]. 

Recently, the synthesis of NPs using biological materials has increased significantly because of the wide range of natural resources and the availability of simple, cost-effective, environmentally friendly, and dependable approaches [36]. In this study, we selected *Evolvulus alsinoides*, which is known as a source of potent antioxidants due to the presence of phenolic compounds [46], as the biological material in our process. Omogbai and Eze [46] demonstrated the presence of secondary metabolites such as glycosides, alkaloids, polyphenols, carbohydrates, amino acids and proteins, saponins, volatile oils, flavonoids, and tannins *in E. alsinoides.* Secondary metabolites of plants appear to serve as potential sources of reducing and stabilizing agents for the synthesis of NPs. Therefore, we have chosen to use unexplored biological materials in the synthesis of PdNPs.

Although numerous studies have reported the synthesis and characterization of PdNPs, the mechanisms of cytotoxicity mediated by biomolecular PdNPs is not well studied. Therefore, this study focused on three main objectives, which were, first, the synthesis and characterization of PdNPs using a simple and environmentally friendly approach with biological materials. Secondly, we investigated the biological activities of as-prepared PdNPs in ovarian cancer A2780 cells and, finally, we investigated the mechanisms mediating the PdNPs-induced cancer cell death. 

## 2. Results and Discussion

### 2.1. Synthesis and Characterization of PdNPs Using UV-Vis Spectroscopy

**Figure 1:**
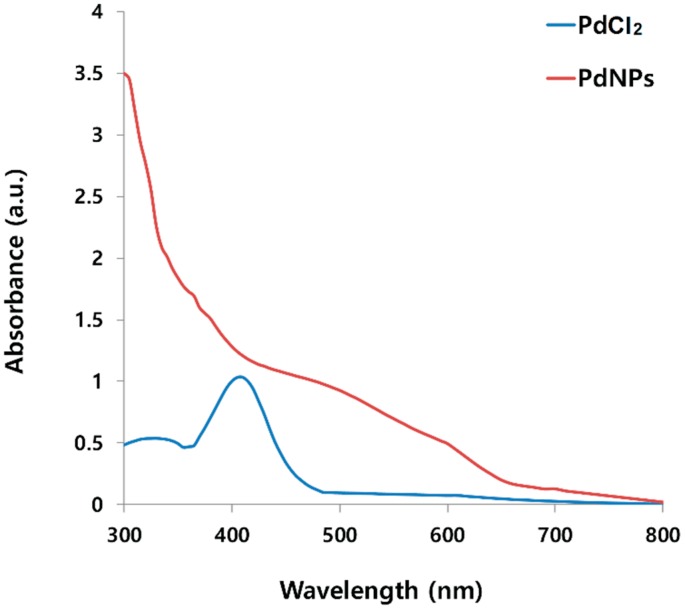
Ultraviolet-Vis (UV-Vis) spectra of aqueous palladium chloride (PdCl_2_) solution and Pd nanoparticles (PdNPs) prepared using leaf extract of *Evolvulus alsinoides*. UV-Vis spectrum of reference sample PdCl_2_ showed a peak at 417 nm due to absorption of Pd(II) ions. For reduced samples, the peak at 417 nm was absent and a broad continuous absorption was observed, indicating formation of PdNPs.

The synthesis of the PdNPs was performed at 60 °C using an aqueous solution of PdCl_2_ and the leaf extract, which was used as a reducing and stabilizing agent. The color of the aqueous solution of PdCl_2_ gradually changed from light yellow to dark brown following the addition of an aqueous solution of the leaf extract with stirring for 2 h at 60 °C, indicating the formation of PdNPs. The synthetic reaction was completed in 6 h. To obtain further evidence of the successful preparation of the PdNPs, we characterized the as-prepared PdNPs. Figure 1 displays the UV-Vis spectra of the PdNPs formed at 60 °C with the reduction of Pd(II) ions to PdNPs mediated by the leaf extract, and the reference sample PdCl_2_ showed a peak at 417 nm due to the absorption of Pd(II) ions. For the reduced samples, the peak at 417 nm was absent, and a broad continuous absorption was observed, indicating a complete reduction of Pd(II) ions to PdNPs. Our results are in agreement with earlier publications including that of Nadagouda and Varma [31], who reported the coffee and tea extract-mediated green synthesis of silver and PdNPs at room temperature. In addition, other studies have also reported similar findings with *Anacardium occidentale* and *Pulicaria glutinosa* leaf extract-mediated PdNP synthesis [43,44].

### 2.2. XRD Analysis of PdNPs

**Figure 2:**
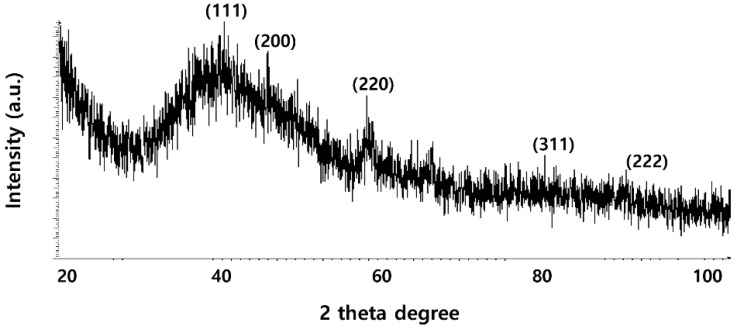
X-ray diffraction (XRD) pattern of dried palladium nanoparticles (PdNPs).

To determine the particle size and crystalline nature of the as-prepared PdNPs, X-ray diffraction (XRD) analysis was performed on dried PdNPs. The results show three characteristic peaks (Figure 2) of PdNPs were observed at 40.0, 46.0, and 63.0° (JCPDS: 87-0643), corresponding to reflections from (1 1 1), (2 0 0) and (2 2 0) planes of the (face-centered cubic) fcc lattice, respectively [43,45,47,48]. The most intensive and predominant peak of the PdNP crystals was observed at 40.0°, corresponding to (1 1 1) planes. The broad peak at 40.0° is the characteristic peak of the (1 1 1) indices of Pd(0), which is a face-centered cubic structure. Considering the full width at half maximum (FWHM) of the (1 1 1) peak, the mean diameter of the NPs was found to be 5 nm, which is in agreement with the results of the TEM studies. Our data were significantly consistent with previous studies using biological material such as coffee and tea extracts [31] as well as the dried leaves of *A. occidentale* [43] and *P. glutinosa* extract [44] as a reducing agent for the synthesis of PdNPs. 

### 2.3. FTIR Analysis of PdNPs

**Figure 3:**
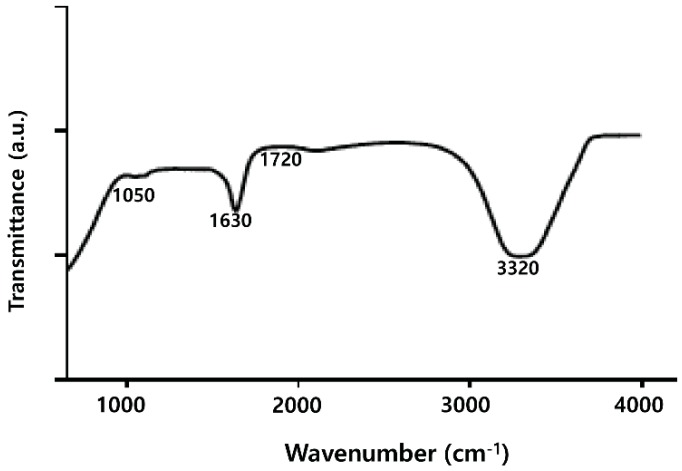
Fourier transform infrared spectroscopy (FTIR) spectra of dried palladium nanoparticles (PdNPs).

FTIR analysis was performed to identify the role of the leaf extract in the reduction and capping of the NPs’ surfaces. The spectra of the leaf extract–stabilized PdNPs are shown in Figure 3. The major absorbance bands were observed at 3320, and 1630 cm^−1^, corresponding to the hydrogen-bonded hydroxyl (OH) and amide I. The absorption peaks present around 1630 cm^−1^ are the characteristic peaks of the C–C stretching of the aromatics [44]. The characteristics peak positions of the –OH stretching vibration, –CO group, vibration at 3320, and 1630, respectively, were observed because the leaf extract facilitated the reduction and stabilization process [44,45]. A similar phenomenon was observed in the synthesis of PdNPs using xanthan gum and leaf extracts of *A. occidentale* [43]. The mechanism of bio-reduction of Pd(II) ions to PdNPs occurs through the secondary metabolites including phenolic compounds and saponins present in leaf extracts binding to nanoparticles via either free amine groups or cysteine residues in proteins. Plant extract of *Evolvulus alsinoides* contains a large amount of natural antioxidants such as alkaloids, tannin, steroids, phenol, saponins, and flavonoids, and these secondary metabolites of *Evolvulus alsinoides* capped the palladium nanoparticles, thereby stabilizing them. Taken together, water-soluble fractions comprised of natural antioxidants in the aqueous plant extract favored the bio-reduction of Pd(II) ions to PdNPs [43,44,45].

### 2.4. DLS Analysis of PdNPs

**Figure 4:**
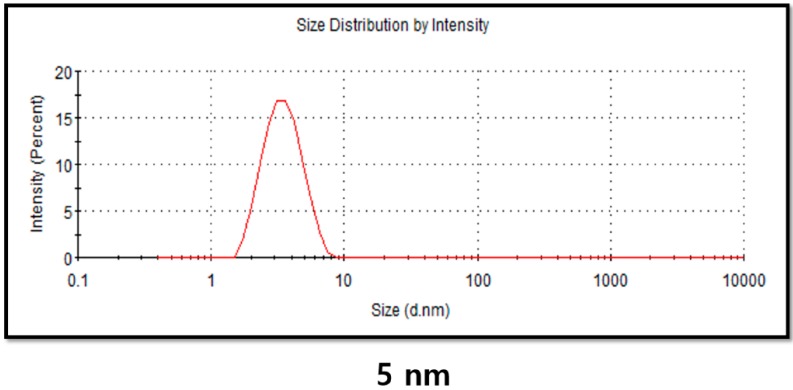
Determination of size distribution of palladium nanoparticles (PdNPs) using dynamic light scattering (DLS).

Particle size, size distribution, morphology, composition, surface area, surface chemistry, and reactivity in solution are important factors essential for assessing NP toxicity [49,50]. Therefore, the physical characterization of any NP synthesized by physical, chemical, and biological methods is necessary before *in vitro* toxicity assessments [49,50,51]. Powers *et al.* [52] explained that Dynamic light scattering (DLS) is a valuable technique for evaluating particle size, size distribution, and the zeta potential of nanomaterials in solution [49,51,52]. DLS measurements were performed in aqueous solution to elucidate the size of PdNPs synthesized by leaf extract biomolecule*.* We found that the average hydrodynamic diameter of the PdNPs was 5 nm (Figure 4). Takeoka *et al.* [53] reported the average diameter of an aqueous dispersion of PdNPs to be 89 nm. The size of the PdNPs determined using the DLS analysis shows a good agreement with sizes obtained from the TEM analysis, with an average size of 5 nm. 

### 2.5. Surface Morphology and Size Analysis of PdNPs

**Figure 5:**
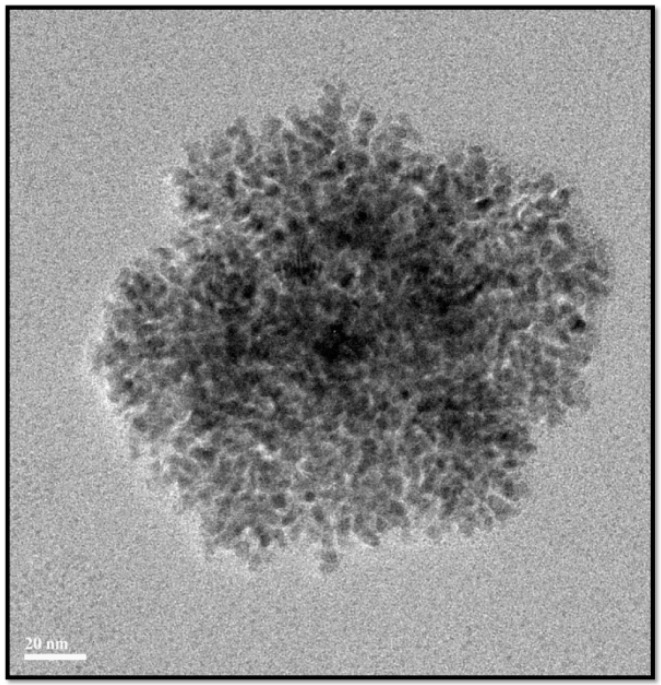
Transmission electron microscopy (TEM) images of palladium nanoparticles (PdNPs).

The surface morphology and size of the as-prepared PdNPs were analyzed using TEM. TEM images of the PdNPs revealed they were spherical in shape and dispersed with an average particle size of 5 nm (Figure 5). Similarly, *A. occidentale* leaf extract produced NPs with a particle size range between 2.5 and 4.5 nm [43]. Qiu *et al.* [54] reported the synthesis of highly dispersed spherical PdNPs and a dendritic Pd superstructure in the presence of cetyltrimethylammonium bromide (CTAB) at room temperature using a simple pulse sono-electrochemical technique. PdNPs with various shapes and sizes can be produced using different biomolecules as reducing agents such as coffee and tea extract, *P. glutinosa* leaf extract, and xanthan gum, with particle sizes of 20–60, 20–25, and 2–12 nm, respectively.

### 2.6. Effect of PdNPs on Viability of Human Ovarian A2780 Cancer Cells 

**Figure 6:**
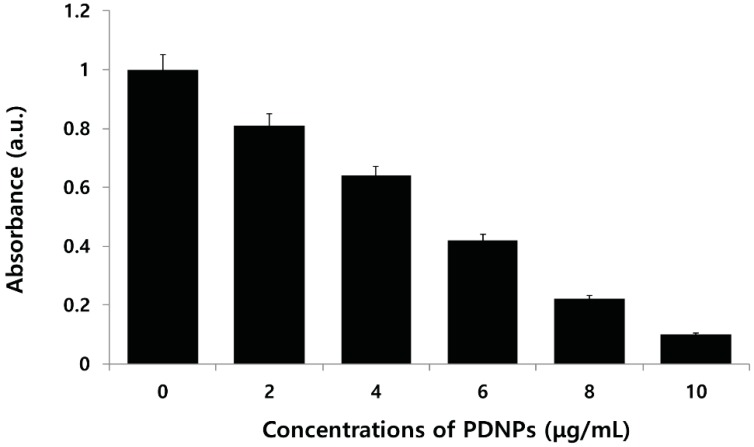
Effects of palladium nanoparticles (PdNPs) on cell viability of human ovarian cancer cells. Viability of A2780 human ovarian cancer cells was determined after 24-h exposure to different concentrations of PdNPs using the water-soluble tetrazolium (WST)-8 assay. Results are mean ± standard deviation of three independent experiments. Significant difference was observed above 4 µg/mL. Viability of treated compared to untreated cells using Student’s *t*-test, *p* < 0.05.

To evaluate the toxicity of PdNPs against ovarian cancer cells, the cells were treated with various concentrations of PdNPs (0–10 μg/mL) and incubated for 24 h. The cell viability was measured using a water-soluble tetrazolium (WST) assay. The results showed that the PdNPs exhibited higher toxicity than the non-treatment of cells did, and dose-dependently inhibited cell viability. Particularly, doses higher than 6 μg/mL induced severe cell death (Figure 6). Fang *et al.* [16] developed a novel drug delivery system (DDS) based on the HMSS–NH_2_@Pd particles, which exhibited a synergistic effect in killing cancer cells. Furthermore, Petrarca *et al.* [55] found that PdNP exposure is associated with an increased percentage of mitogen activation with a diploid DNA content, indicative of the maintenance of the G0 state or prolongation/arrest in the G1-phase in human peripheral blood mononuclear cells (PBMCs). In addition, Yusop *et al.* [56] demonstrated that polystyrene-coated PdNPs induced cell damage via cell cycle disturbance by the withdrawal or inactivation of nutrients, phytohemagglutinin-l (PHA-l), or both, or by modulation or inhibition of downstream signaling due to their catalytic activity [55,56,57]. Wilkinson *et al.* [25] demonstrated that PdNPs do not affect the viability of human primary bronchial epithelial cells (PBEC) or A549 cells at doses up to 10 μg/mL. However, PdNPs affect cell viability at higher doses. Interestingly, PBEC were markedly more affected by PdNPs than A549 cells were. Long *et al.* [58] observed that Pd nanocubes (NCs) significantly reduced the viability of HeLa cells. Taken together, the results suggest that inhibition of cell viability by PdNPs is dose-dependent.

### 2.7. Effect of PdNPs on Cell Morphology

**Figure 7:**
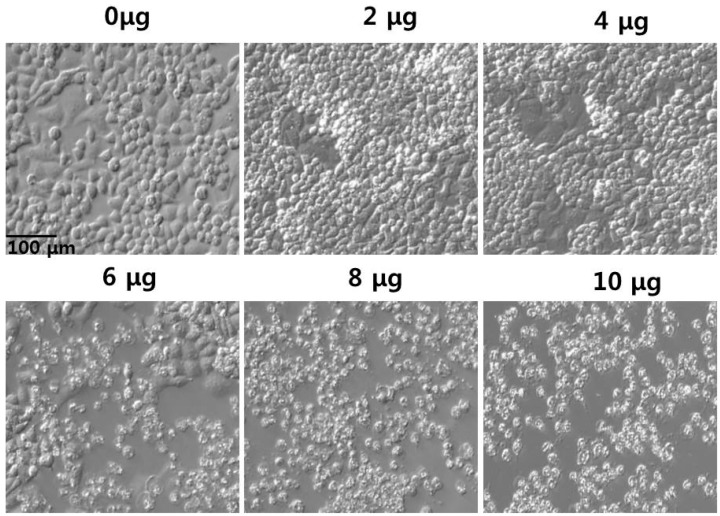
Morphology of human ovarian cancer cells treated with palladium nanoparticles (PdNPs) Morphology of A2780 cells treated with various concentrations of PdNPs (0–10 μg/mL) for 24 h. Images were recorded using interference contrast light microscopy.

To corroborate the results of the cell viability assay, we examined the effect of PdNPs on cell morphology. The cells were incubated with PdNPs, which exhibited significant toxicity at the tested concentrations. The results show that at 6–10 μg/mL, the PdNPs induced noticeable morphological changes, which were more severe with increasing concentrations of PdNPs (Figure 7). In addition, the cells exhibited shrunken cell membranes and debris was observed in the cancer cells treated with 10 μg/mL for 24 h, indicating that the cellular morphology was significantly different from that of the control sample. Furthermore, the cells were sensitive to PdNPs, which altered the viability and shape. Interestingly, the cell density decreased more in the PdNP-treated groups than it did in untreated groups. 

### 2.8. Effect of PdNPs on LDH Leakage

**Figure 8:**
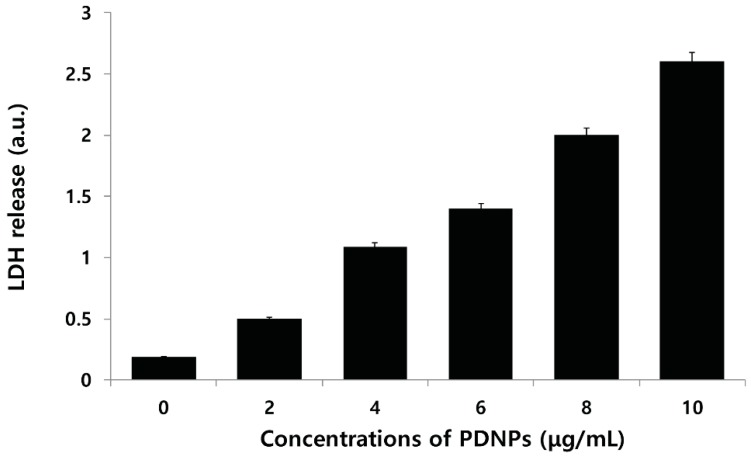
Effect of palladium nanoparticles (PdNPs) on leakage of lactate dehydrogenase (LDH). LDH activity was measured at 490 nm using an LDH cytotoxicity kit. Results are mean ± standard deviation (SD) of three independent experiments. Significant difference in LDH activity of PdNPs compared to untreated cells was evident using Student’s *t*-test, *p* < 0.05.

To determine whether the inhibitory effect of PdNPs on cell viability is related to the induction of apoptosis, ovarian cancer cells were incubated with increasing concentrations of the PdNPs for 24 h and intracellular LDH release as a result of the breakdown of the plasma membrane and the alteration of its permeability was evaluated. The LDH assay is a widely recognized cytotoxicity endpoint for the measurement of cell membrane integrity and viability [59]. The results suggest that compared to untreated control cells, PdNPs produced a concentration-dependent increase in LDH leakage when incubated with cancer cells (Figure 8). Therefore, we demonstrated that PdNPs exhibited dose-dependent cytotoxic effects against ovarian cancer cells, by inducing apoptosis as a specific feature. 

### 2.9. Effect of PdNPs on ROS Generation

**Figure 9:**
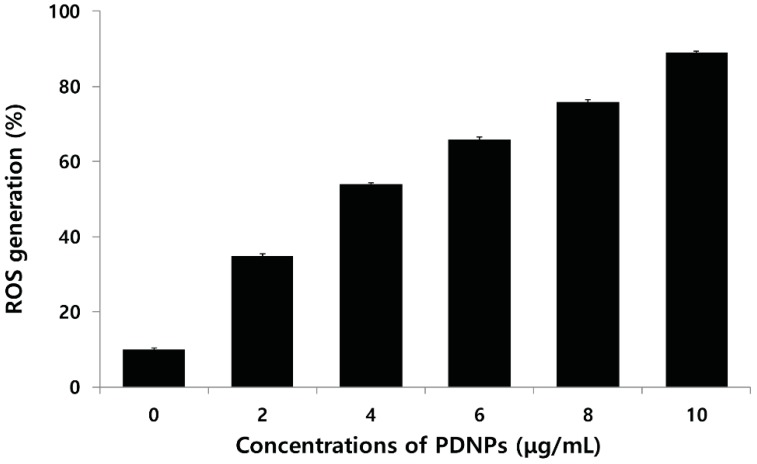
Palladium nanoparticles (PdNPs) induce reactive oxygen species (ROS) generation in human ovarian cancer cells. Relative fluorescence of 2′,7′-dichlorofluorescein was measured at excitation and emission wavelengths of 485 and 530 nm, respectively, using a spectrofluorometer. Results are mean ± standard deviation (SD) of three independent experiments. Treated groups showed statistically significant differences from control group using Student’s *t*-test, *p* < 0.05.

The generation of reactive oxygen species (ROS) leads to oxidative stress, which in turn may trigger cell death. To support the evidence obtained from the LDH assay, ROS was measured ovarian cancer cells treated by PdNPs for 24 h by monitoring the enzymatic cleavage of dichlorodihydrofluorescein diacetate (DCFH-DA) expressed as a percentage of the untreated control relative to ROS levels. ROS generation was significantly higher in the PdNP-treated cells than it was in the untreated controls, and the effect was dose-dependent (Figure 9). Xia *et al.* [60] explained the cytotoxicity of certain NPs based on their propensity to induce oxidative stress. For example, different NPs are known to induce oxidative stress, including silver, gold, and silica NPs in breast cancer [61], Burkitt lymphoma B and epithelial breast cancer [62], and HepG2 cells [63] respectively. However, there are no significant studies available on PdNP-induced cell death via oxidative stress, particularly in ovarian cancer cells. Recently, Wilkinson *et al.* [25] demonstrated that PdNPs induced apoptosis dose-dependently in PBEC but not in A549 cells. In addition, they observed that 25 μg/mL PdNPs induced caspase-3-like enzyme activity in PBEC, as evidenced in the DEVD-AMC assay. Long *et al.* [58] reported that PdNPs efficiently generated singlet oxygen radicals via simple chemisorption of molecular oxygen on their surface without the process being facilitated by light. Neubauer *et al.* [64] demonstrated ROS production by Pd and Ni NPs. The results of previous studies revealed that Ni exhibited a linear dependent ROS production at catalyst concentration in the range of 4–250 μg/mL. ROS production by Pd was significantly higher than it was by Ni. The surface-specific ROS production activities of both Pd and Ni showed a pronounced dependence on the particle size being in the range of 4–27 nm, with maximum activity in the vicinity of 12 nm for both acellular and cellular environments. These results suggest that smaller-sized PdNPs induce higher levels of ROS than larger particles do.

### 2.10. PdNP-Induced Autophagy and Autophagic Cell Death

**Figure 10:**
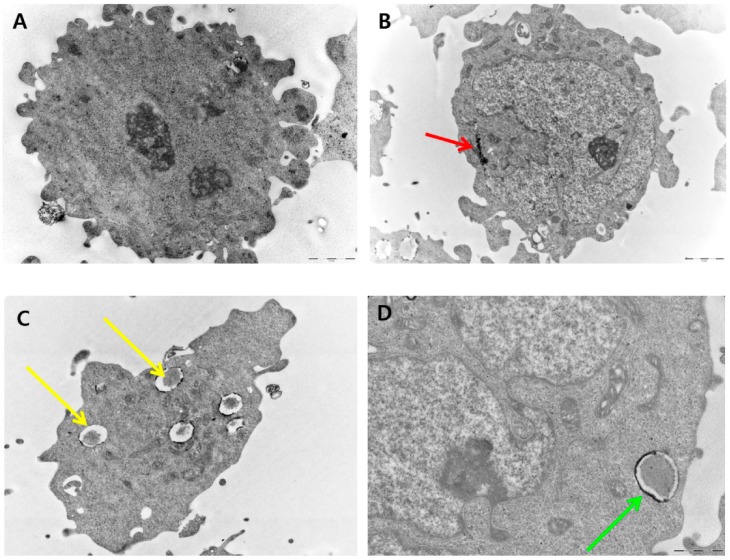

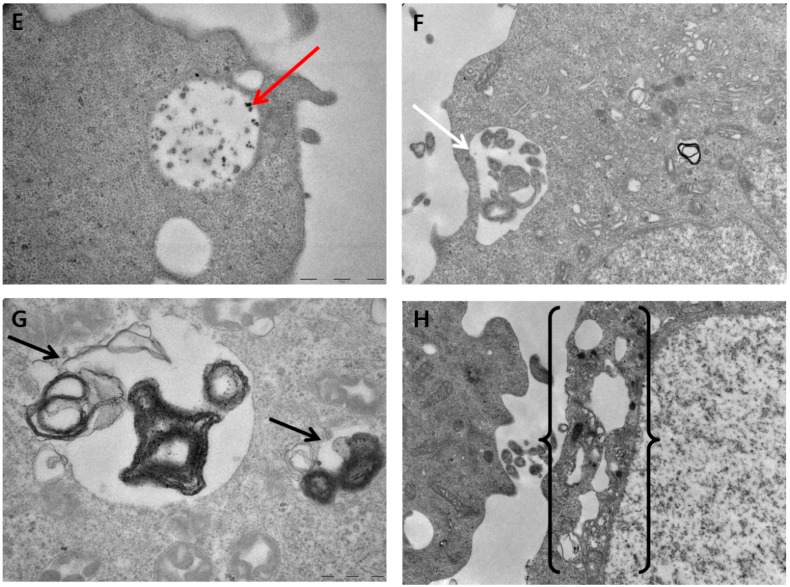
Palladium nanoparticles (PdNPs) induce autophagy and autophagic cell death. Autophagic ultrastructural features in human ovarian cancer cells exposed to 10 μg/mL of PdNPs. (**A**) Control cells show typical distribution of organelles; (**B**) Internalization of PdNPs in cytoplasm of cells (red arrow); (**C**) Double-membranous phagophores developing into autophagosomes (yellow arrows); (**D**) Autophagosome (green arrow); (**E**) Internalization of PdNPs in lysosomes (red arrow); (**F**) Autolysosomes containing dead cell with electron-lucent cytoplasm and destroyed organelles, with membrane-bound cytoplasmic material (white arrow); (**G**) Fusion of autophagosomes with lysosomes or endosomes (black arrow); (**H**) Autophagic vacuoles containing membrane-bound cytoplasmic material (long bracket).

Various stress-related conditions can activate autophagy such as starvation, oxidative stress, heat, and infection [65]. Recently, numerous studies have suggested that the mitochondria may produce massive amounts of ROS that can modulate autophagic processes [66]. We hypothesized that the excessive generation of ROS induced by PdNPs could trigger autophagy and eventually lead to autophagic cell death. To clarify the mechanisms of the PdNP-induced autophagy, we investigated this phenomenon in ovarian cancer cells. Autophagy involves the sequestration and degradation of cargo materials including cellular components, organelles, and proteins by the lysosomal machinery for energy recycling and to maintain homeostasis [63,67,68,69]. Zabirnyk *et al.* [69] reported that nanomaterials have been recognized as a novel class of autophagy activators because they could be perceived by cells as the endosomal pathogens or proteins, which are commonly degraded by the autophagy pathway, and could induce autophagy dysfunction, leading to severe pathological states [70]. Autophagic dysfunction leads to excessive autophagy induction or blockade of autophagic flux, which eventually leads to induction of potential mechanisms of cell death [70]. To elucidate the mechanisms mediating autophagic cell death induced by PdNPs, internalization and further consequences to the cells were determined using TEM. The images of the control cells show clear morphology with distinct nuclei (Figure 10A). The cells treated with PdNPs exhibit internalization of PdNPs into the cytoplasm (Figure 10B), which induced the formation of numerous autophagosomes (Figure 10C). Interestingly, the PdNPs did not only induce autophagosomes but were also localized in the autophagosomes and autolysosomes (Figure 10D,E). As shown in Figure 10B–E, the PdNPs penetrated the cells, induced autophagy of the ovarian cancer cells following exposure for 24 h, and accumulated in the lysosomes (Figure 10E). In addition, the PdNPs induced the formation of autolysosomes, which contain dead cell with electron-lucent cytoplasm and damaged organelles (Figure 10F,G). Our data are consistent with a previous report showing that the cytotoxicity of NPs was linked closely to their lysosomal localization in both normal and cancer cells [71]. Autophagic cell death is considered to be the cell death that is triggered by autophagy and occurred accompanied by massive autophagic vacuolization of the cytoplasm [63] (Figure 10H). Collectively, the results of this study showed that the activation of autophagy was accompanied by an increase in ROS levels in cells exposed to PdNPs.

### 2.11. Effect of PdNPs on Mitochondrial Membrane Potential (MMP)

**Figure 11:**
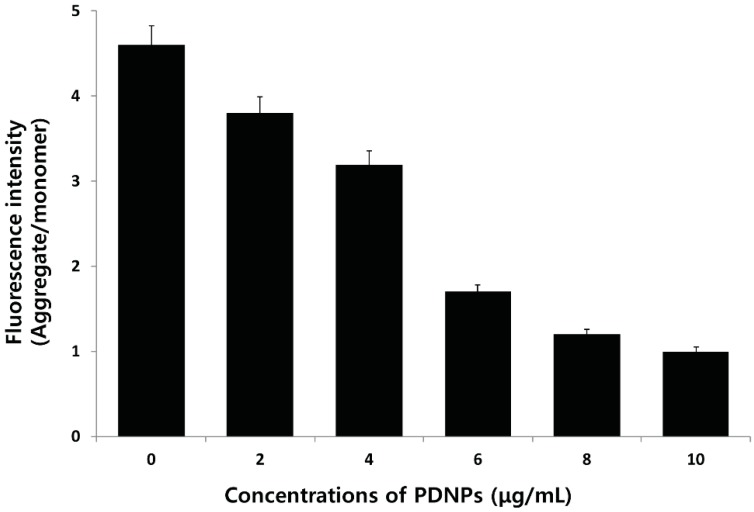
Effect of palladium nanoparticles (PdNPs) on mitochondrial membrane potential (MMP). MMP (ratio of JC1 aggregate to monomer) in ovarian cancer cells determined after treatment with different concentrations of PdNPs for 24 h.

Numerous studies have clearly indicated that the mode of cell death depends on the severity of the cellular insult, which may in turn be linked to mitochondrial function and intracellular energy [72]. Mitochondria are the major site of intracellular ROS production, which occurs via electron leakage as a byproduct of adenosine triphosphate (ATP) generation by oxidative phosphorylation, and programmed cell death [73]. The depolarization of the mitochondrial membrane is the initial event in apoptosis induction in the presence of PdNPs. To determine whether the loss of mitochondrial membrane potential (MMP) occurred in PdNP-treated cells, we used JC-1, a mitochondrial dye that stains mitochondria in living cells in a membrane potential–dependent fashion. The cells were exposed to different concentrations of PdNPs, incubated with JC-1 dye, and then the fluorescence was measured using a spectrofluorometer. After a 24 h of PdNP exposure, a dramatic dose-dependent decrease in the ratio of red-green fluorescence intensity could be observed, indicating rapid depolarization of the mitochondrial membranes. Significant depolarization with a greater than five-fold decrease in the ΔΨ_m_ could be observed after treatment with PdNPs (Figure 11). These results suggest that the collapse of the ΔΨ_m_ is an early event in PdNP-induced apoptosis [74]. Control cells showed a JC-1 red-green fluorescence intensity ratio that was higher than that of the PdNP-treated cells. In contrast, treatment with PdNPs depolarized the mitochondrial membrane in ovarian cancer cells, as evidenced by the loss of the JC-1 red-green fluorescence intensity, indicating a loss of mitochondrial membrane integrity. Similarly, we previously observed a loss of MMP in AgNPs-treated MDA-MB-231 human breast cancer [75] and lung carcinoma (A549) cells [76]. 

Previous studies suggest that the mitochondria play a key role in the apoptotic cell death pathway, and changes in mitochondrial membrane permeability comprise the early events of apoptosis via depolarization of the mitochondrial membrane [77,78,79,80]. Depolarized mitochondria result from the formation of mitochondrial permeability transition pores (MPTPs). Mitochondrial PT has been associated with various metabolic events such as halted functioning of the electron transport chain with associated elevation in ROS and decreased production of ATP [81]. The present results indicate that adverse changes in the mitochondrial function induced by PdNPs trigger apoptosis, which is possibly associated with intracellular ROS production. Our results are in agreement with previous reports in the literature and suggest that an increase in ROS generation after exposure to PdNPs disrupts the mitochondrial membrane and induces apoptosis. Freyre-Fonseca *et al.* [82] demonstrated that TiO_2_NPs exposure induces alterations in the mitochondria including cytochrome c release into the cytosol, mitochondrial permeability changes, and decreased MMP. 

### 2.12. Effect of PdNPs on MDA Content and Antioxidant Enzyme Activities

The cytotoxicity caused by ROS normally accompanies an increase in lipid peroxides [83]. The oxidation of membrane lipids, one of the primary events in oxidative cellular damage, can be assessed by measuring MDA, a breakdown product of lipid peroxides [83]. Constant exposure of cells to free radicals could lead to the development of complex defense mechanisms involving enzymatic and non-enzymatic antioxidants. Several antioxidant enzymes play an important role in the detoxification process to prevent cell damage caused by oxidative stress and other types of stress caused by exogenous factors such as NPs [80,81,82,84]. Complex organisms have natural defense mechanisms such as antioxidative and repair systems to reduce the oxidative damage. Therefore, we investigated whether PdNPs play oxidative or anti-oxidative roles by treating cells with different concentrations and measuring both MDA and antioxidant activities including Superoxide dismutase (SOD) and Catalase (CAT). Treatment of ovarian cells with different concentration of PdNPs (0–10 μg/mL) significantly increased the intracellular MDA level (Table 1), indicating that PdNPs may potentially induce oxidative damage in cells. In addition, PdNPs increased the MDA level dose-dependently. Similarly, Niska *et al.* [84] demonstrated that TiO_2_NPs enhanced the production of superoxide anions and altered the antioxidant system of human osteoblast cells. 

**Table 1:** Effect of palladium nanoparticles (PdNPs) on malondialdehyde (MDA), superoxide dismutase (SOD), and catalase (CAT) activities. A2780 cells were treated with various concentrations of PdNPs for 24 h. Then cells were harvested and washed twice with an ice-cold PBS solution. Cells were collected, homogenized, and homogenate was centrifuged at 4 °C at 10,000 rpm for 15 min. Concentration of MDA and activities of SOD and CAT were measured.

Treatment	MDA (nmole/mg Protein)	SOD (U/mg Protein)	CAT (U/mg Protein)
Control	0.51 + 0.02	60.7 + 2.1	45.66 + 2.3
2 μg/mL	1.21 + 0.05 *	50.34 + 3.3 *	26.63 + 4.4 *
4 μg/mL	1.61 + 0.08 *	45.1 + 3.8 *	16.11 + 2.9 *
6 μg/mL	2.10 +0.04 *	34.0 + 2.2 *	8.1 + 8.1 *
8 μg/mL	2.61 + 0.09 *	22.12 + 1.6 *	4.0 + 1.1 *
10 μg/mL	2.80 + 0.03 *	12.34 + 4.5 *	2.0 + 1.4 *

* Significant difference (*p* < 0.05) between treated groups and control.

Furthermore, to determine the effects on antioxidant systems, we measured SOD and CAT activities and found that incubation of ovarian cells with PdNPs significantly reduced their levels (Table 1). SOD is an enzyme that primarily catalyzes the dismutation of superoxide radicals and CAT catalyzes the decomposition of H_2_O_2_. The cells treated with PdNPs showed a decline of SOD and CAT activity and the antioxidant activities were dose-dependent. These data suggest that the failure of the antioxidant system to restore the redox equilibrium in the cells following exposure to PdNPs could lead to cellular damage and death.

### 2.13. Effect of PdNPs on Capsase-3 Activity

ROS plays an important role in triggering cell damage including the formation of mitochondrial permeability transition pore (MPTP) that leads to the activation of mitochondrial-dependent cell death pathways [85,86]. The caspases are a family of intracellular cysteine-dependent, aspartate-specific proteases, which reside as latent precursors in most cells and propagate cell death [72]. Mitochondria are the center of cell metabolism and energy transformation, and their malfunction decreases cell viability [87]. The primary feature of disruption of Δψ_m_ appears to be a point of no return in the Fas-induced apoptosis of Jurkat cells, secondary to the activation of caspases [88,89].

**Figure 12:**
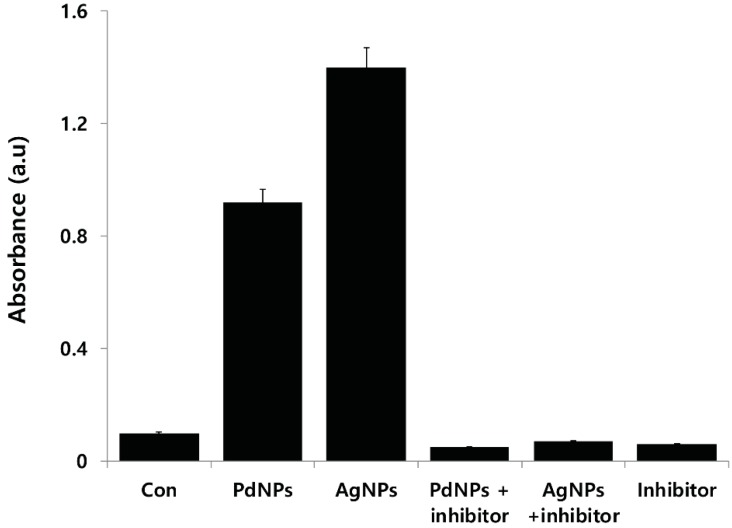
Effect of palladium nanoparticles (PdNPs) on caspase-3 activity in human ovarian cancer cells. Ovarian cancer cells were treated with 10 μg/mL of PdNPs with or without caspase-3 inhibitor (Ac-DEVD-CHO) for 24 h. Concentration of *p*-nitroanilide released from substrate was calculated from absorbance at 405 nm. Results are mean ± standard deviation (SD) of three separate experiments. Treated groups found significant differences from control group using Student’s *t*-test, *p* < 0.05.

Caspase-3 is a protease known to be involved in apoptotic cell death. In agreement with this notion, we determined the effect of PdNPs on caspase-3 activation. To see the expression level of caspase-3, the cells were treated with PdNPs (10 μg/mL) for 24 h. The results show that in PdNP-treated cells, caspase-3 activity increased significantly compared to that of the untreated control cells (Figure 12). Ovarian cancer cells exposed to PdNPs at concentrations of 10 μg/mL showed increased caspase-3 expressions, whereas pretreatment with a caspase inhibitor completely inhibited this effect, which indicates that the activation of caspase-3 was absolutely required for cell death. The present study suggests that impairment of the MMP increased ROS production, which is consistent with the notion that degenerated mitochondria are the primary site of ROS production [90]. Alarifi *et al.* [91] reported that iron oxide NPs caused the death of MCF-7 cells, which was mediated by the ROS-triggered mitochondrial pathway, as evidenced by the cleavage of the caspase-3 activity. Taken together, these results suggest that mitochondrial impairment is a primary event in the toxicity induced by PdNPs in ovarian cancer cells.

### 2.14. PdNPs Induces Apoptosis

**Figure 13:**
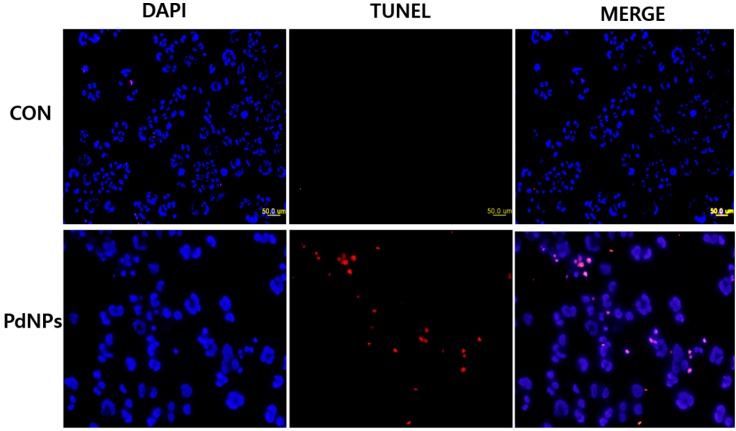
Palladium nanoparticles (PdNPs) induce apoptosis of human ovarian cancer cells. Human ovarian cancer cells were treated with 10 μg/mL PdNPs for 24 h and apoptosis was assessed using terminal deoxynucleotidyl transferase dUTP nick end labeling (TUNEL) assay. Nuclei were counterstained with 4′,6-diamidino-2-phenylindole (DAPI). Representative images show apoptotic (fragmented), DNA (red staining), and corresponding nuclei (blue staining) (Scale bar-50 µm).

Apoptotic cell death has been implicated in various disease states [92]. Caspases are crucial components of the cell death pathways and caspase-3 is activated by numerous death signals and cleaves a variety of important cellular proteins, which are required for DNA fragmentation and some of the typical morphological changes in cells undergoing apoptosis [93]. DNA fragmentation is one of the most important final and irreversible events in apoptosis [94]. Enari *et al.* [95] demonstrated the link between caspase activation and nuclear DNA fragmentation using caspase-activated DNase (CAD). In addition, DNA fragmentation is a hallmark of apoptosis. Therefore, we sought to determine whether PdNP-induced caspase-3 activation is involved in DNA fragmentation. Human ovarian cells were exposed to 10 μg/mL of PdNPs for 24 h and then the TUNEL analysis was performed. The results indicate that treatment with PdNPs caused the appearance of a significant number of TUNEL-positive cells, whereas no apoptotic cells were observed in the untreated controls (Figure 13). Recently, Takaki *et al.* [96] showed that TiO2 NPs induced apoptosis associated with DNA fragmentation and caspase-3 activation. Our observation of DNA fragmentation after 24 h of PdNP treatment is consistent with the significant increase in caspase-3 activity after 24 h of PdNP treatment. These results suggest that PdNPs induce DNA fragmentation by caspase-3 activation. Wilkinson *et al.* [25] demonstrated that PdNPs (10 nm) induced cell death in human PBEC but not A549 cells at the doses tested (up to 25 μg/mL) via activation of caspase-3. Numerous recent studies investigated the potential toxicity of PdNPs and their adverse health effects such as concentration-dependent cytotoxicity, induction of apoptosis, and alterations of the release and expression of numerous cytokines [19,20,22,23,24,25]. Taken together, the results of our study suggest that PdNPs induce cell death via caspase-3 activation and DNA fragmentation; however, the degree of toxicity is dependent on the physical and chemical properties of the synthesized PdNPs.

## 3. Materials and Methods

### 3.1. Materials

PdCl_2_ for the preparation of the PdNPs was purchased from Sigma-Aldrich (St. Louis, MO, USA). Penicillin-streptomycin solution, trypsin-ethylenediaminetetraacetic acid (EDTA) solution, Dulbecco’s modified Eagle’s medium (DMEM, F-12), and 1% antibiotic-antimycotic solution were obtained from Life Technologies (Carlsbad, CA, USA). A fetal bovine serum (FBS) *in vitro* toxicology assay kit was purchased from Sigma-Aldrich. The reagent kits for the measurement of malondialdehyde (MDA), superoxide dismutase (SOD), and catalase (CAT) were purchased from Sigma-Aldrich.

### 3.2. Synthesis of PdNPs

The PdNPs were prepared using *E. alsinoides* plant extracts according to a previously described method [44]. *E. alsinoides* leaves were collected and stored at 4 °C until required. Then, 24 g was washed thoroughly with double-distilled water, cut into fine pieces of approximately 1–5 cm using a sharp stainless steel knife, suspended in 100 mL of sterile distilled water, and then boiled for 5 min. The resulting mixture was filtered through a Whatman filter paper no. 1. The filtered extract was used for the synthesis of PdNPs by adding 10 mL to 100 mL of 1 mM aqueous PdCl_2_ solution, and then incubated for 6 h at 60 °C with constant stirring. The reduction occurred rapidly and was indicated by a color change in the solution from light to bright brown. The Pd colloid formed is stable at room temperature (26 °C) for more than three months.

### 3.3. Characterization of PdNPs 

The ultraviolet-visible (UV-Vis) spectra of the PdNPs were recorded using an Optizen Pop spectrophotometer (Mecasys, Seoul, Korea) while X-ray diffraction (XRD) analyses were performed using a Bruker D8 Discover X-ray diffractometer (Bruker AXS GmBH, Karlsruhe, Germany). The X-ray source was 3 kW with a Cu target, and high-resolution XRD patterns were measured using a scintillation counter (*λ* = 1.5406 Å). The XRD was run at 40 kV and 40 mA, and samples were recorded at 2θ values between 10° and 80°. The dried PdNP powder was diluted with potassium bromide and Fourier transform infrared spectroscopy (FTIR, Perkin Elmer Inc., Waltham, MA, USA) and GX spectrometry were performed, and the spectra were recorded within the range of 500–4000 cm^−1^. Transmission electron microscopy (TEM) using a TEM Hitachi H-7500 (Seoul National University, Seoul, Korea) was used to determine the PdNP size and morphology with images obtained at an accelerating voltage of 300 kV. 

### 3.4. Cell Culture and Exposure to PdNPs

Human ovarian A2780 cancer cells were cultured in DMEM supplemented with 10% FBS, 2 mM glutamine, and 100 U/mL penicillin-streptomycin in a humidified 5% CO_2_ incubator at 37 °C. The medium was replaced three times a week, and cells were passaged at subconfluency. At approximately 75% confluence, cells were harvested using 0.25% trypsin-EDTA and seeded in 75 cm^2^ flasks, six-well, or 96-well plates depending on the experiment. After 24 h, the medium was replaced with fresh medium containing PdNPs at different concentrations (0–10 µg/mL) while cells not exposed to PdNPs served as the negative control. After a 24-h incubation, the treated cells were analyzed for viability, lactate dehydrogenase (LDH) release, and reactive oxygen species (ROS) generation while cell morphology was examined using an Olympus IX71 microscope (Tokyo, Japan) with appropriate filter sets.

### 3.5. Cell Viability Assay

Cell viability was examined using the WST-8 assay as described previously [35,76]. Briefly, 1 × 10^4^ cells were seeded in 100 μL of 10% FBS in DMEM in a 96-well plate. After 24 h, the cells were washed with 100 μL of serum-free DMEM twice and incubated in 100 μL of serum-free DMEM containing different concentrations of the PdNP suspensions. After a 24-h exposure, the cells were washed twice with serum-free DMEM, and 15 μL of the WST-8 solution was added to each well containing 100 μL of serum-free DMEM. After a 1-h incubation, 80 μL of the mixture was transferred to another 96-well plate to avoid the possible effect of residual PdNPs on the absorbance, which was measured at 450 nm using a microplate reader. Cell-free control experiments were performed to determine whether the PdNPs directly reacted with the WST-8 reagent by incubating various concentrations of resveratrol (0–200 µM) or 100 μL of PdNP suspensions (0–10 μg/mL) with 10 μL of the WST-8 reagent in a 96-well plate for 1 h. The plates were centrifuged to precipitate the PdNPs, and 100 μL of the supernatant was transferred to another 96-well plate to measure the optical density at 450 nm.

### 3.6. Cell Morphology

Ovarian cancer cells were plated in six-well plates (1 × 10^4^ cells/well) in 10% FBS in DMEM. After 24 h, the medium was changed to serum-free DMEM without or with various concentrations (0–10 μg/mL) of PdNPs and incubated for 24 h. Cell morphology was examined using an optical microscope.

### 3.7. Membrane Integrity

The cell membrane integrity of human ovarian cancer cells (A2780) was evaluated by determining LDH activity in cell supernatants using an *In Vitro* Toxicology Assay kit (Sigma-Aldrich) according to the manufacturer’s instructions and as described previously [76]. Briefly, cells were exposed to various concentrations of PdNPs (0–10 μg/mL) in triplicate for 24 h, 100 μL of each cell-free supernatant was transferred to a new 96-well plate, and mixed with 100 μL of the LDH reagent. After a 3-h incubation under standard conditions, the optical density was determined at 490 nm using a microplate reader.

### 3.8. Determination of ROS 

Intracellular ROS was measured based on the intracellular peroxide-dependent oxidation of 2′,7′-dichlorodihydrofluorescein diacetate (DCFH-DA, Molecular Probes, Eugene, OR, USA) to a fluorescent compound 2′,7′-dichlorofluorescein (DCF) as previously described [61]. Cells were seeded into 24-well plates at a density of 5 × 10^4^ cells/well and cultured for 24 h. After washing twice with PBS, fresh medium containing different concentrations of PdNPs (0–10 μg/mL) was added and the cells were incubated for 24 h. The cells were then supplemented with 20 μM DCFH-DA and incubation continued for 30 min at 37 °C. The cells were rinsed with PBS, and then 2 mL of PBS was added to each well, and fluorescence intensity was determined using a Gemini EM spectrofluorometer (Molecular Devices, Sunnyvale, CA, USA) with the excitation at 485 nm and emission at 530 nm. 

### 3.9. TEM Analysis

TEM analysis was used to detect whether autophagy was induced in PdNPs-treated human ovarian cancer cells. After ovarian cancer cells had been incubated for 24 h with PdNPs (10 μg/mL), the cells were washed with PBS and then centrifuged at 1500 rpm for 10 min. The supernatants were removed, the cell pellets were fixed with 2.5% glutaraldehyde (Ted Pella Inc., 18426, Redding, CA, USA) in phosphate-buffered saline (PBS, 0.1 M, pH 7.2) for 3 h, post-fixed in 1% osmium tetroxide (Heraeus, 89.740.219, Hanau, Germany), dehydrated in a graded series of ethanol baths and propylene oxide (Acros Organics, 149620025, Fair Lawn, NJ, USA), and then embedded in epoxy resin (EMbed 812 containing nadic methyl anhydride, dodecenyl succinic anhydride, and DMP-30) as described previously [30]. Serial ultrathin sections were cut using an LKB Ultratome III (Leica, Wetzlar, Germany), stained with uranyl acetate and lead citrate (Ted Pella Inc., 19485 and 19314, respectively), and examined under a Hitachi H7600 electron microscope (TEM; Hitachi H-7500, Hitachi Ltd., Tokyo, Japan) at an accelerating voltage of 100 kV.

### 3.10. Mitochondrial Membrane Potential (MMP)

The mitochondrial membrane potential (MMP) was measured as described previously [23,74,97] using a cationic fluorescent indicator JC-1 (Molecular Probes). JC-1 is a lipophilic cation, which, in a reaction driven by ΔΨ_m_ in normal polarized mitochondria, assembles into a red fluorescence-emitting dimer forming JC-1-aggregates. However, the monomeric form present in cells with depolarized mitochondrial membranes emits only green fluorescence. JC1-aggregates in intact mitochondria exhibit a red fluorescence with an emission at 583 nm while JC1-monomers in the cytoplasm show green fluorescence with an emission and excitation at 525 and 488 nm wavelengths. Cells were incubated with 10 μM JC-1 at 37 °C for 15 min, washed with PBS, resuspended in PBS, and then the fluorescence intensity was measured. MMP was expressed as the ratio of the fluorescence intensity of the JC1 aggregates to monomers. 

### 3.11. Measurement of MDA Content and Antioxidant Enzyme Activities

The MDA content and antioxidant enzyme activities were measured using a method described previously [83]. Ovarian cancer cells were treated with different concentrations of PdNPs for 24 h, and then the MDA content was determined using the thiobarbituric acid method. Antioxidant enzyme activities were assayed after cells were incubated with different concentrations of PdNPs for 24 h. The measurements were performed according to the manufacturer’s instructions for the reagent kits. For MDA and antioxidant enzyme assays, the cell cultures were washed twice with PBS, scraped from the plates into ice-cold PBS (0.1 M, containing 0.05 mM EDTA), and homogenized. The homogenate was centrifuged at 4 °C at 10,000 rpm for 15 min, and the resulting supernatant was stored at 70 °C until analyzed. Protein concentration was determined using the Bradford method with bovine serum albumin (BSA) as a reference standard.

### 3.12. Measurement of Caspase-3 Activity

Caspase-3 activity was assayed as described previously [61,98,99] using a commercial kit (Sigma-Aldrich) according to the manufacturer’s instructions. Cancer cells were plated as described above, treated without or with PdNPs (10 μg/mL) or a caspase-3 inhibitor for 24 h, washed with ice-cold PBS, and then lysed with 100 μL of lysis buffer containing 50 mM Tris-hydrochloride (HCl), pH 7.5, 150 mM sodium chloride (NaCl), 1 mM ethylene glycol tetraacetic acid (EGTA), 1 mM sodium fluoride (NaF), 1% Nonidet P-40, 1 mM phenylmethanesulfonyl fluoride (PMSF), and protease inhibitor cocktail for 30 min at 4 °C. The extracts were collected by centrifugation at 10,000 rpm for 10 min and the protein concentration was determined using the Bio-Rad protein assay kit (Bio-Rad, Hercules, CA, USA). Equal amounts (50 μg) of protein extracts were mixed with an assay buffer containing 20 mM HEPES (pH 7.4), 100 mM NaCl, 0.1% 3-([3-cholamidopropyl]dimethylammonio)-1-propanesulfonate (CHAPS), 10 mM dithiothreitol (DTT), 1 mM EDTA, and 10% sucrose added to 96-well plates, incubated with the caspase-3 substrate and inhibitor acetyl-Asp-Glu-Val-Asp *p*-nitroanilide (Ac-DEVD-pNA) and Ac-DEVD-CHO, respectively, for 1 h, and then the absorbance was measured at 405 nm. The colorimetric assay is based on the hydrolysis of the caspase-3 substrate by caspase-3, resulting in the release of pNA. 

### 3.13. Terminal Deoxynucleotidyl Transferase dUTP Nick End Labeling (TUNEL) Assay

PdNP-induced apoptosis was determined using a terminal deoxynucleotidyl transferase dUTP nick end labeling (TUNEL) assay using the DNA fragmentation Imaging kit (Roche) following the manufacturer’s instruction and also the method described previously [98]. Briefly, A2780 cells were seeded in six-well plates (1.5 × 10^6^ cells/well) for 24 h, and then treated with 10 μg/mL PdNPs for another 24 h. The cells were then detached with trypsin-EDTA, placed on 0.01% polylysine-coated slides, fixed with 4% methanol-free formaldehyde solution, and stained using terminal deoxynucleotidyltransferase and fluorescein-labeled dUTP for fluorescence-based detection of cells containing DNA breaks. The cells were counterstained with 4′,6-diamidino-2-phenylindole (DAPI) to determine the total cell number. Stained cells were observed using a Carl-Zeiss epifluorescence microscope (Olympus, Tokyo, Japan) equipped with a triple band-pass filter. To determine the percentage of apoptotic cells, 1000 cells were counted in each experiment. Merged images of TUNEL- and DAPI-stained cells were observed using a fluorescence microscope (Olympus) at ×500 magnification. 

### 3.14. Statistical Analyses

All the assays were conducted in triplicate, and experiments were repeated at least thrice. The results are presented as means ± standard deviation (SD). All the experimental data were analyzed using the Student’s *t*-test. A *p*-value < 0.05 was considered statistically significant.

## 4. Conclusions

The main focus of the NP studies currently being carried out is to develop tools for the targeted delivery of drugs, sensing, and energy storage, as well as to formulate contrasting materials, food additives, and catalysis. In particular, PdNPs have been studied and used as very efficient catalysts for the reactions of various compounds. In this study, we described the synthesis of PdNPs using leaf extracts of *E. alsinoides* without using any additional chemical agents for reduction or stabilization. This green chemistry approach appears to be simple, cost-effective, and dependable for the production of PdNPs. Furthermore, the apoptotic potential of the PdNPs was evaluated in ovarian cancer cells. The efficiency of *in vitro* toxicity was evident from the suppression of cell viability, increased leakage of LDH, elevation of ROS generation, induction of autophagy and autophagic cell death, and enhanced caspase-3 activity followed by DNA fragmentation. Impairment of the MMP was primarily caused by the ROS induced by PdNPs, which further potentiated oxidative stress and cell death. Therefore, we conclude that PdNPs induce caspase-dependent apoptosis. The substantial anticancer effect of the PdNPs warrants future investigations of similar PdNPs for development as potential anticancer therapies. Further studies demonstrating the detailed mechanisms and therapeutic potential of PdNPs in both *in vitro* and *in vivo* animal models of cancer diseases are required. 
